# Supplementary material for: A Novel Piggyback Strategy for mRNA Delivery Exploiting Adenovirus Entry Biology
Source: Viruses. 2022 Sep 30;14(10):2169. doi: 10.3390/v14102169 (PMC9608319; doi:10.3390/v14102169)
Supplement: Supplementary file 1 [file viruses-14-02169-s001.zip › viruses-1909106-supplementary material-updated.pdf]

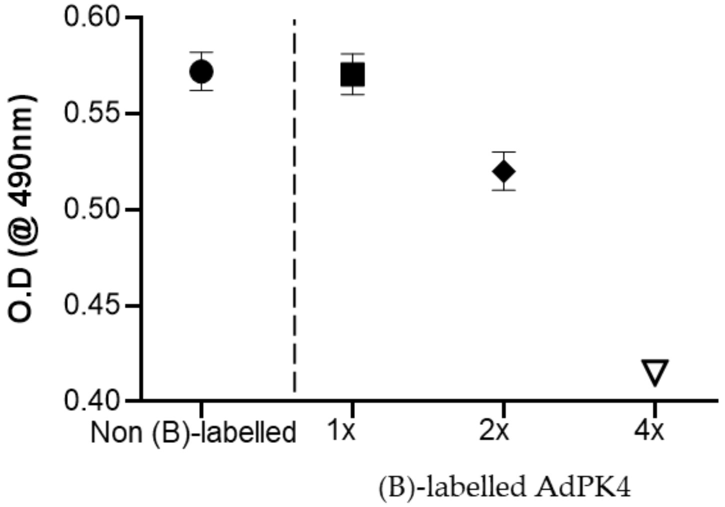

Figure S1. Validation of AdPK4-STAVpLys-mRNA complexes. Verification of biotinylation of AdPK4.CMV.GFP was determined through HABA assay by measuring absorbance with different amounts of virus (1x, 2x and 4x) at A490-500 nm. The non-biotinylated adenovirus was also assessed as a control. 1X, 2X, and 4X correspond to 1×10^{10}, 2×10^{10}, and 4×10^{10} viral particles, respectively.
